# Ethyl (*Z*)-2-chloro-2-[2-(4-meth­oxy­phenyl)hydrazin-1-yl­idene]acetate

**DOI:** 10.1107/S1600536812044285

**Published:** 2012-11-03

**Authors:** Abdullah M. Asiri, Muhammad Nadeem Arshad, Mohie E. M. Zayed, Khalid A. Alamry, Muhammad Shafiq

**Affiliations:** a Chemistry Department and the, Center of Excellence for Advanced Materials Research (CEAMR), Faculty of Science, King Abdulaziz University, PO Box 80203, Jeddah 21589, Saudi Arabia; b Center of Excellence for Advanced Materials Research (CEAMR), Faculty of Science, King Abdulaziz University, PO Box 80203, Jeddah 21589, Saudi Arabia; c Chemistry Department, Faculty of Science, King Abdulaziz University, PO Box 80203, Jeddah 21589, Saudi Arabia; d Department of Chemistry, Government College University, 38000, Faisalabad, Pakistan

## Abstract

The mol­ecule of the title compound, C_11_H_13_ClN_2_O_3_, is planar (r.m.s. deviation = 0.0587 Å for non-H atoms) and adopts a *Z* conformation about the C=N double bond. In the crystal, mol­ecules are linked *via* an N—H⋯O hydrogen bond, forming zigzag chains propagating along [010]. These chains are consolidated by C—H⋯O hydrogen bonds.

## Related literature

For closely related structures, see: Asiri *et al.* (2011*a* ▶,*b* ▶). For graph-set notation, see: Bernstein *et al.* (1995 ▶).
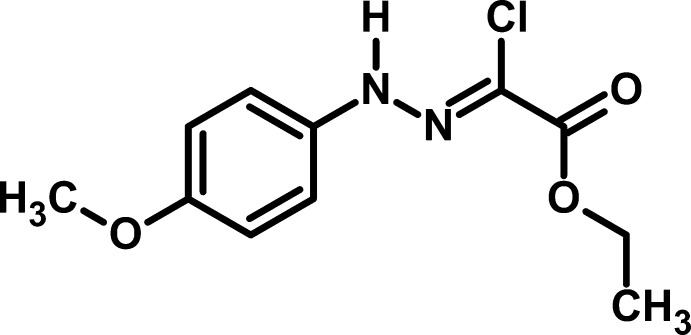


## Experimental

### 

#### Crystal data

C_11_H_13_ClN_2_O_3_
*M* *_r_* = 256.68
Monoclinic, 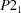

*a* = 4.7480 (2) Å
*b* = 9.9256 (4) Å
*c* = 13.3084 (4) Å
β = 91.468 (3)°
*V* = 626.98 (4) Å^3^
*Z* = 2
Cu *K*α radiation
μ = 2.71 mm^−1^
*T* = 296 K
0.23 × 0.11 × 0.06 mm

#### Data collection

Agilent SuperNova (Dual, Cu at zero, Atlas) CCD diffractometer
Absorption correction: multi-scan (*CrysAlis PRO*; Agilent, 2012 ▶) *T*_min_ = 0.860, *T*_max_ = 1.000
3153 measured reflections
1824 independent reflections
1685 reflections with *I* > 2σ(*I*)
*R*_int_ = 0.018

#### Refinement

*R*[*F* ^2^ > 2σ(*F* ^2^)] = 0.038
*wR*(*F* ^2^) = 0.109
*S* = 1.07
1824 reflections
159 parameters
1 restraint
H atoms treated by a mixture of independent and constrained refinement
Δρ_max_ = 0.16 e Å^−3^
Δρ_min_ = −0.20 e Å^−3^
Absolute structure: Flack (1983 ▶), 466 Friedel pairs
Flack parameter: 0.01 (2)

### 

Data collection: *CrysAlis PRO* (Agilent, 2012 ▶); cell refinement: *CrysAlis PRO*; data reduction: *CrysAlis PRO*; program(s) used to solve structure: *SHELXS97* (Sheldrick, 2008 ▶); program(s) used to refine structure: *SHELXL97* (Sheldrick, 2008 ▶); molecular graphics: *PLATON* (Spek, 2009 ▶); software used to prepare material for publication: *WinGX* (Farrugia, 2012 ▶).

## Supplementary Material

Crystal structure: contains datablock(s) I, global. DOI: 10.1107/S1600536812044285/su2515sup1.cif

Structure factors: contains datablock(s) I. DOI: 10.1107/S1600536812044285/su2515Isup2.hkl

Supplementary material file. DOI: 10.1107/S1600536812044285/su2515Isup3.cml

Additional supplementary materials:  crystallographic information; 3D view; checkCIF report

## Figures and Tables

**Table 1:** Hydrogen-bond geometry (Å, °)

*D*—H⋯*A*	*D*—H	H⋯*A*	*D*⋯*A*	*D*—H⋯*A*
N1—H1*N*⋯O2^i^	0.97 (4)	2.11 (4)	3.053 (3)	164 (3)
C6—H6⋯O2^i^	0.93	2.59	3.368 (3)	141

Symmetry code: (i) 
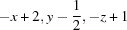
.

**Fig. 1.**
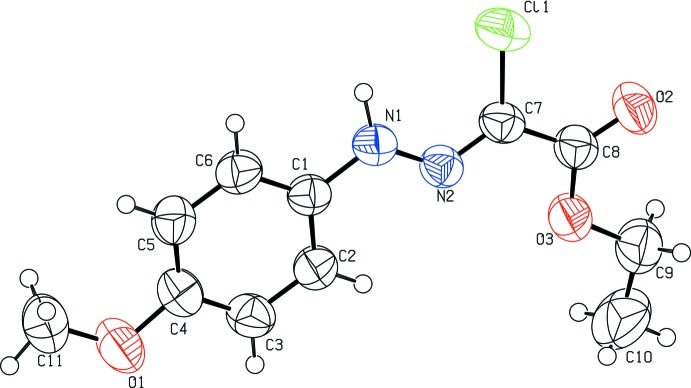
A view of the molecular structure of the title molecule with the atom numbering. The displacement ellipsoids are drawn at the 50% probability level.

**Fig. 2.**
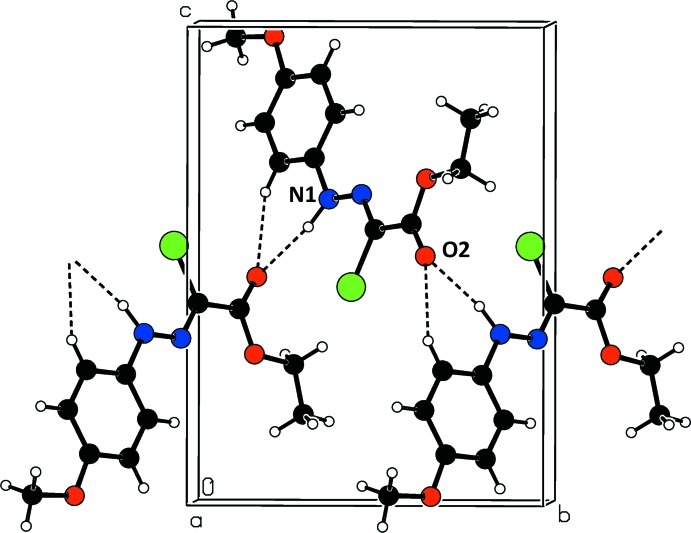
A partial view along the a axis of the crystal packing of the title compound. The N-H···O and C-H···O hydrogen bonds are shown as dashed lines - see Table 1 for details.

## supplementary crystallographic information

### Comment

The present structure analysis is a continuation of our interest in related compounds already reported by our group, that is, 1-Chloro-1-[(4-methoxyphenyl)hydrazinylidene]propan-2-one (Asiri *et al.*, 2011*a*) and 1-Chloro-1-[(4-methylphenyl)hydrazinylidene]propan-2-one (Asiri *et al.*, 2011*b*).

In the title compound, Fig. 1, the methoxy aromatic ring (C1—C6) is oriented at a dihedral angle of 3.05 (2) ° with respect to the mean plane of the ester moiety (N1/N2/C7-Cl1; planar to within 0.0 \%A). The molecule adopts a Z conformation around the C7═N2 double bond.

In the crystal, N-H···O and C-H···O hydrogen bonds connect the molecules to form zigzag chains along the *b* axis, enclosing six membered *R*^1^_2_(6) ring motifs (Bernstein *et al.*, 1995) - see Table 1 and Fig. 2.

### Experimental

The molecule was synthesised according to the literature procedure (Asiri *et al.*, 2011*a*) and recrystallized from ethanol giving yellow needle-like crystals.

### Refinement

The NH H atom was located in a difference Fourier map and refined with *U*_iso_(H) = 1.2U_eq_(N). The C-bound H-atoms were included in calculated positions and treated as riding atoms: C-H = 0.93, 0.96 and 0.97 Å for CH(aromatic), CH_3_, and CH_2_ H atoms, respectively, with U_iso_(H) = k × U_eq_(parent C-atom), where k = 1.5 for CH_3_ H atoms and = 1.2 for other H atoms.

### Crystal data

**Table:**

C_11_H_13_ClN_2_O_3_	*F*(000) = 268
*M**_r_* = 256.68	*D*_x_ = 1.360 Mg m^−^^3^
Monoclinic, *P*2_1_	Cu *K*α radiation, λ = 1.54184 Å
Hall symbol: P 2yb	Cell parameters from 2064 reflections
*a* = 4.7480 (2) Å	θ = 4.5–75.6°
*b* = 9.9256 (4) Å	µ = 2.71 mm^−^^1^
*c* = 13.3084 (4) Å	*T* = 296 K
β = 91.468 (3)°	Needle, yellow
*V* = 626.98 (4) Å^3^	0.23 × 0.11 × 0.06 mm
*Z* = 2	

### Data collection

**Table:**

Agilent SuperNova (Dual, Cu at zero, Atlas) CCD diffractometer	1824 independent reflections
Radiation source: SuperNova (Cu) X-ray Source	1685 reflections with *I* > 2σ(*I*)
Mirror monochromator	*R*_int_ = 0.018
ω scans	θ_max_ = 75.8°, θ_min_ = 5.6°
Absorption correction: multi-scan (*CrysAlis PRO*; Agilent, 2012)	*h* = −5→5
*T*_min_ = 0.860, *T*_max_ = 1.000	*k* = −12→9
3153 measured reflections	*l* = −16→16

### Refinement

**Table:**

Refinement on *F*^2^	Secondary atom site location: difference Fourier map
Least-squares matrix: full	Hydrogen site location: inferred from neighbouring sites
*R*[*F*^2^ > 2σ(*F*^2^)] = 0.038	H atoms treated by a mixture of independent and constrained refinement
*wR*(*F*^2^) = 0.109	*w* = 1/[σ^2^(*F*_o_^2^) + (0.0575*P*)^2^ + 0.0434*P*] where *P* = (*F*_o_^2^ + 2*F*_c_^2^)/3
*S* = 1.07	(Δ/σ)_max_ < 0.001
1824 reflections	Δρ_max_ = 0.16 e Å^−^^3^
159 parameters	Δρ_min_ = −0.20 e Å^−^^3^
1 restraint	Absolute structure: Flack (1983), 466 Friedel pairs
Primary atom site location: structure-invariant direct methods	Flack parameter: 0.01 (2)

### Special details

**Table:**

Geometry. All esds (except the esd in the dihedral angle between two l.s. planes) are estimated using the full covariance matrix. The cell esds are taken into account individually in the estimation of esds in distances, angles and torsion angles; correlations between esds in cell parameters are only used when they are defined by crystal symmetry. An approximate (isotropic) treatment of cell esds is used for estimating esds involving l.s. planes.
Refinement. Refinement of F^2^ against ALL reflections. The weighted R-factor wR and goodness of fit S are based on F^2^, conventional R-factors R are based on F, with F set to zero for negative F^2^. The threshold expression of F^2^ > 2sigma(F^2^) is used only for calculating R-factors(gt) etc. and is not relevant to the choice of reflections for refinement. R-factors based on F^2^ are statistically about twice as large as those based on F, and R- factors based on ALL data will be even larger.

### Fractional atomic coordinates and isotropic or equivalent isotropic displacement parameters (Å^2^)

**Table:**

	*x*	*y*	*z*	*U*_iso_*/*U*_eq_	
Cl1	1.03853 (17)	0.46169 (11)	0.45757 (5)	0.0873 (3)	
O1	0.1393 (5)	0.2136 (3)	0.96956 (16)	0.0895 (7)	
O2	1.4090 (4)	0.6817 (2)	0.52714 (16)	0.0785 (6)	
O3	1.2764 (5)	0.6800 (2)	0.68691 (15)	0.0735 (6)	
N1	0.7239 (5)	0.3882 (3)	0.63734 (15)	0.0607 (5)	
N2	0.9085 (4)	0.4860 (2)	0.64964 (14)	0.0566 (5)	
C1	0.5738 (5)	0.3420 (3)	0.72120 (18)	0.0542 (5)	
C2	0.6135 (6)	0.4022 (3)	0.81488 (19)	0.0616 (6)	
H2	0.7405	0.4728	0.8235	0.074*	
C3	0.4620 (6)	0.3557 (3)	0.89470 (19)	0.0675 (7)	
H3	0.4869	0.3960	0.9574	0.081*	
C4	0.2750 (6)	0.2509 (3)	0.8836 (2)	0.0656 (7)	
C5	0.2349 (6)	0.1908 (3)	0.7907 (2)	0.0666 (7)	
H5	0.1083	0.1199	0.7824	0.080*	
C6	0.3865 (6)	0.2378 (3)	0.7097 (2)	0.0637 (7)	
H6	0.3604	0.1979	0.6469	0.076*	
C7	1.0590 (6)	0.5288 (3)	0.57810 (18)	0.0584 (6)	
C8	1.2652 (6)	0.6375 (3)	0.59250 (18)	0.0605 (6)	
C9	1.4822 (8)	0.7834 (4)	0.7110 (2)	0.0894 (10)	
H9A	1.6681	0.7533	0.6923	0.107*	
H9B	1.4376	0.8647	0.6734	0.107*	
C10	1.4804 (13)	0.8107 (5)	0.8152 (3)	0.1310 (18)	
H10A	1.2976	0.8430	0.8329	0.197*	
H10B	1.6195	0.8780	0.8315	0.197*	
H10C	1.5228	0.7297	0.8520	0.197*	
C11	−0.0364 (8)	0.1011 (4)	0.9655 (3)	0.0903 (11)	
H11A	−0.1846	0.1157	0.9162	0.135*	
H11B	−0.1166	0.0869	1.0301	0.135*	
H11C	0.0709	0.0233	0.9473	0.135*	
H1N	0.712 (7)	0.330 (5)	0.579 (3)	0.108*	

### Atomic displacement parameters (Å^2^)

**Table:**

	*U*^11^	*U*^22^	*U*^33^	*U*^12^	*U*^13^	*U*^23^
Cl1	0.1022 (6)	0.1004 (6)	0.0602 (4)	−0.0065 (5)	0.0182 (3)	−0.0155 (4)
O1	0.0895 (14)	0.112 (2)	0.0679 (12)	−0.0237 (15)	0.0142 (10)	0.0084 (13)
O2	0.0887 (14)	0.0810 (15)	0.0666 (11)	−0.0067 (12)	0.0164 (9)	0.0181 (10)
O3	0.0847 (13)	0.0763 (13)	0.0597 (10)	−0.0197 (11)	0.0073 (9)	0.0004 (9)
N1	0.0667 (12)	0.0627 (13)	0.0528 (11)	−0.0039 (11)	0.0032 (9)	−0.0050 (9)
N2	0.0595 (11)	0.0586 (14)	0.0518 (9)	0.0038 (10)	0.0020 (8)	0.0034 (9)
C1	0.0548 (13)	0.0542 (13)	0.0537 (12)	0.0056 (11)	0.0003 (9)	−0.0011 (10)
C2	0.0642 (14)	0.0615 (15)	0.0591 (14)	−0.0054 (13)	0.0007 (10)	−0.0026 (11)
C3	0.0701 (16)	0.0773 (19)	0.0551 (13)	−0.0018 (15)	0.0021 (11)	−0.0052 (13)
C4	0.0605 (14)	0.0745 (18)	0.0619 (15)	0.0016 (14)	0.0031 (11)	0.0094 (13)
C5	0.0634 (15)	0.0655 (17)	0.0708 (15)	−0.0077 (14)	−0.0009 (11)	0.0018 (13)
C6	0.0685 (16)	0.0640 (16)	0.0583 (14)	−0.0046 (14)	−0.0035 (11)	−0.0065 (12)
C7	0.0618 (14)	0.0601 (15)	0.0533 (12)	0.0101 (12)	0.0053 (10)	0.0017 (10)
C8	0.0687 (15)	0.0577 (15)	0.0554 (12)	0.0069 (13)	0.0042 (10)	0.0079 (11)
C9	0.106 (3)	0.083 (2)	0.0788 (19)	−0.026 (2)	0.0017 (17)	0.0007 (17)
C10	0.191 (5)	0.103 (3)	0.098 (3)	−0.042 (4)	−0.014 (3)	−0.020 (3)
C11	0.081 (2)	0.088 (3)	0.103 (2)	−0.006 (2)	0.0184 (18)	0.020 (2)

### Geometric parameters (Å, º)

**Table:**

Cl1—C7	1.737 (3)	C3—H3	0.9300
O1—C4	1.378 (3)	C4—C5	1.381 (4)
O1—C11	1.394 (5)	C5—C6	1.393 (4)
O2—C8	1.202 (3)	C5—H5	0.9300
O3—C8	1.325 (3)	C6—H6	0.9300
O3—C9	1.447 (4)	C7—C8	1.466 (4)
N1—N2	1.315 (3)	C9—C10	1.414 (5)
N1—C1	1.416 (3)	C9—H9A	0.9700
N1—H1N	0.97 (4)	C9—H9B	0.9700
N2—C7	1.277 (3)	C10—H10A	0.9600
C1—C6	1.370 (4)	C10—H10B	0.9600
C1—C2	1.391 (3)	C10—H10C	0.9600
C2—C3	1.378 (4)	C11—H11A	0.9600
C2—H2	0.9300	C11—H11B	0.9600
C3—C4	1.373 (4)	C11—H11C	0.9600
			
C4—O1—C11	118.3 (3)	N2—C7—C8	122.1 (2)
C8—O3—C9	116.5 (2)	N2—C7—Cl1	122.8 (2)
N2—N1—C1	119.3 (2)	C8—C7—Cl1	115.10 (19)
N2—N1—H1N	124 (2)	O2—C8—O3	124.1 (3)
C1—N1—H1N	115 (2)	O2—C8—C7	124.3 (3)
C7—N2—N1	122.4 (2)	O3—C8—C7	111.6 (2)
C6—C1—C2	119.8 (2)	C10—C9—O3	109.4 (3)
C6—C1—N1	119.7 (2)	C10—C9—H9A	109.8
C2—C1—N1	120.5 (3)	O3—C9—H9A	109.8
C3—C2—C1	119.1 (3)	C10—C9—H9B	109.8
C3—C2—H2	120.5	O3—C9—H9B	109.8
C1—C2—H2	120.5	H9A—C9—H9B	108.2
C4—C3—C2	121.3 (2)	C9—C10—H10A	109.5
C4—C3—H3	119.4	C9—C10—H10B	109.5
C2—C3—H3	119.4	H10A—C10—H10B	109.5
C3—C4—O1	115.4 (3)	C9—C10—H10C	109.5
C3—C4—C5	119.9 (2)	H10A—C10—H10C	109.5
O1—C4—C5	124.8 (3)	H10B—C10—H10C	109.5
C4—C5—C6	119.1 (3)	O1—C11—H11A	109.5
C4—C5—H5	120.5	O1—C11—H11B	109.5
C6—C5—H5	120.5	H11A—C11—H11B	109.5
C1—C6—C5	120.9 (2)	O1—C11—H11C	109.5
C1—C6—H6	119.6	H11A—C11—H11C	109.5
C5—C6—H6	119.6	H11B—C11—H11C	109.5
			
C1—N1—N2—C7	177.1 (2)	C2—C1—C6—C5	−0.1 (4)
N2—N1—C1—C6	−178.3 (2)	N1—C1—C6—C5	−179.6 (3)
N2—N1—C1—C2	2.2 (4)	C4—C5—C6—C1	0.1 (4)
C6—C1—C2—C3	−0.2 (4)	N1—N2—C7—C8	−179.7 (2)
N1—C1—C2—C3	179.4 (3)	N1—N2—C7—Cl1	−0.6 (4)
C1—C2—C3—C4	0.5 (5)	C9—O3—C8—O2	−1.8 (4)
C2—C3—C4—O1	179.4 (3)	C9—O3—C8—C7	177.3 (3)
C2—C3—C4—C5	−0.4 (5)	N2—C7—C8—O2	−179.3 (3)
C11—O1—C4—C3	−175.0 (3)	Cl1—C7—C8—O2	1.5 (4)
C11—O1—C4—C5	4.9 (5)	N2—C7—C8—O3	1.6 (4)
C3—C4—C5—C6	0.1 (5)	Cl1—C7—C8—O3	−177.6 (2)
O1—C4—C5—C6	−179.7 (3)	C8—O3—C9—C10	−176.1 (4)

### Hydrogen-bond geometry (Å, º)

**Table:**

*D*—H···*A*	*D*—H	H···*A*	*D*···*A*	*D*—H···*A*
N1—H1*N*···O2^i^	0.97 (4)	2.11 (4)	3.053 (3)	164 (3)
C6—H6···O2^i^	0.93	2.59	3.368 (3)	141

Symmetry code: (i) −*x*+2, *y*−1/2, −*z*+1.
